# Performance management methods and practices among nurses in primary health care settings: a systematic scoping review protocol

**DOI:** 10.1186/s13643-020-01294-w

**Published:** 2020-02-21

**Authors:** Cynthia Zandile Madlabana, Tivani Phosa Mashamba-Thompson, Inge Petersen

**Affiliations:** 1 School of Applied Human Sciences, Discipline of Psychology, University of KwaZulu-Natal, Durban, 4001 Republic of South Africa; 2 Discipline of Public Health, University of KwaZulu-Natal, Durban, 4001 Republic of South Africa

**Keywords:** Performance management, Performance appraisals, Performance review, Nurses, Quality of care, Human resources management, Systematic scoping review, Primary health care

## Abstract

**Background:**

Nurses make up the largest constituent of the health workforce. The success of health care interventions depends on nurses’ ability and willingness to provide quality health care services. A well-implemented performance management (PM) system can be a valuable asset in ensuring that nurses are motivated, promoted, trained and rewarded appropriately. Despite the significant benefits of effective PM such as improved motivation, job satisfaction and morale, PM systems are highly contested. Therefore, it is important to examine evidence on PM methods and practices in order to understand its consequences among nursing professionals in primary health care (PHC) settings.

**Methods:**

The search strategy of this systematic scoping review will involve various electronic databases which include *Academic Search Complete*, *PsycARTICLES*. *PsycINFO*, *Cumulative Index to Nursing and Applied Health Literature*, *Medline* and *Cochrane Library* from the EbsocHost Database Platform. Electronic databases such as *PubMed* and *Google Scholar,* Union catalogue of theses and dissertations via SABINET online and WorldCat dissertations will be incorporated. A grey literature search will be conducted on websites such as the *World Health Organization* and *government websites* to find relevant policies and guidelines. The period for the search is from 1978 to 2018. This time period was chosen to coincide with the Declaration of Alma-Ata on PHC adopted in 1978. All references will be exported to Endnote library. Two independent reviewers will begin screening for eligible titles, abstracts and full articles. During title and abstract screening, duplicates will be removed. The Mixed Method Appraisal Tool will determine the quality of included studies. Thematic analysis will be used to analyse the included articles.

**Discussion:**

Evidence of preferences on PM methods and practices will generate insight on the use of PM systems in PHC and how this can be used for the purpose of improving nurses’ performance and in turn, the provision of quality health care. We hope to expose knowledge gaps and inform future research.

## Background

Growing health challenges have placed pressure on health management to monitor and evaluate human resource for health (HRH) in an effort to strengthen health systems response to evolving health challenges [1]. One such challenge is chronic conditions. Chronic conditions present the largest public health challenge of the twenty-first century [2]. It is projected that by 2020, heart disease, stroke, depression and cancer will be the greatest contributors of non-communicable diseases (NCDs), with mental disorders accounting for 60% of total mortality in the world. The number of people that require daily health care is rapidly growing, and it is projected that NCDs will continue to increase at a higher rate in lower-socio economic groups [2]. This has created a need for NCDs surveillance, prevention and control [3]. If not managed appropriately, chronic multiple NCDs will become the most expensive problem faced by health care systems globally [3]. This has resulted in the need for the re-organisation of health care systems to cater for chronic conditions, with people-centred care identified as the optimal approach to cater for multimorbid chronic conditions [4]. Noticeably, the ability of a country to strengthen its health system in order to meet its health goals depends largely on its human capital [5]. The six core components or ‘building blocks’ of the World Health Organization (WHO)’s analytical framework of health systems includes the health workforce as the people responsible for organising and delivering quality health services [6, 7]. Quality health care refers to services that consistently deliver care that improves or maintains health, is valued and trusted by recipients and is responsive to changing population needs [5, 6], with people-centred services identified as central to this endeavour globally given the changing disease profile towards chronic multimorbidity (see Table 1 for definition of quality care). In order to achieve the above, the health workforce must possess the knowledge, skills, motivation and preparedness to engage in actions with the primary intent to improve the provision of quality health services for people-centred services. Therefore, it is of vital importance that health workers are motivated and supported with the relevant capacities, thereby ensuring that they significantly contribute to attaining health objectives set nationally and globally [6, 7]. One of the key human resource (HR) processes used to facilitate training and motivation of any workforce is a performance management (PM) system.

**Table 1:** Key concepts and definitions

Concept	Definition	Example (s)
PM methods	PM methods refer to the particular procedures, processes or tools used to consolidate data on the performance of staff, in the case of this study, registered nurses.	- Conducting annual performance appraisals through 360 ° feedback, peer reviews, behaviourally anchored rating scales (BARS) and critical incidents.
PM practices	PM practices refer to actual application and use of a PM method, as opposed to theories relating to it.	- The above- mentioned methods are known to be effective in providing performance data; however, in practice, factors such as the users’ attitude towards PM methods, training of rater and ratees as well as the provision of constructive performance feedback may impact on the overall success of a PM system.
Quality health care/quality of care	The WHO definition of quality of care is the extent to which health care services provided to individuals and communities improve desired health outcomes. Therefore, in order to achieve ‘quality of care’, health care must be safe, effectively, timely, efficient, equitable and people-centred.	- ‘Safe: Delivering health care that minimizes risks and harm to service users, including avoiding preventable injuries and reducing medical errors. - Effective: Providing services based on scientific knowledge and evidence-based guidelines. - Timely: Reducing delays in providing and receiving health care. - Efficient: Delivering health care in a manner that maximizes resource use and avoids waste. - Equitable: Delivering health care that does not differ in quality according to personal characteristics such as gender, race, ethnicity, geographical location or socioeconomic status. - People-centred: Providing care that takes into account the preferences and aspirations of individual service users and the culture of their community.’ [8]
According to the PCC framework: The targeted **population** for this review is nurses/nurse practitioners/registered nurses.	Nurses for the purpose of this review refer to nurse practitioners/advanced practice nurses (also referred to as registered nurses). The characteristics of this kind of nurse are shaped by the context and/or country that s/he is credentialed to practice.	For instance, a registered nurse must have acquired the necessary expert knowledge base to work in an environment that requires complex decision-making and clinical competencies for expanded practice. Such nurses can generally work independently in clinics and private practices as primary health care providers.
**Concept (s):** Performance management/performance appraisal/performance review	The concept of performance management (for this review) refers to a process of monitoring, reviewing and appraising registered nurses’ work performance over a certain period of time.	- The purpose of this process is to ensure accountability; thus, it would assist nurse managers in administering incentives towards good performance (eg. a performance bonus) and identify gap in one’s performance so to develop/improve one’s ability (training and development needs). The terms performance management, appraisal or review are used to describe this process—the preferred term to use is based on the context/country in which it is used.
**Context:** Primary health care	Primary health care refers to health care provided in the community for people initialling a visit to a medical practitioner or clinic for advice/treatment.	- The review is concerned with nurses that work in the primary health care setting. These nurses work at clinics, community-based health care centres, general practices and home-based health services.

PM is described as a continuous process to identify, measure and improve the performance of individuals, teams and organisation, which involves aligning performance activities with the strategic goals of the organisation [7]. An important component of a PM system is performance appraisal (PA). PA refers to the formal process of assessing performance at work. PA is also sometimes referred to as performance review [9].

Accordingly, PA is a necessary component of PM systems. Some researchers argue that due to previous research not distinguishing between these two concepts, these terms are generally used interchangeably [9, 10]. This study will do the same.

Accordingly, PM systems primarily serve three broad functions:

i. Strategically, PM systems aim to achieve the strategic objectives of the organisation, which is achieved by linking the organisation’s goals with individual performance goals [11].
ii. Administratively, PM provides essential information to help managers take important decisions regarding salary increments, promotions and rewards [12].
iii. The developmental function is facilitated through the provision of feedback on evaluated performance. Through the feedback mechanism, remedial action and steps to improve performance should be discussed. This presents an opportunity for managers to coach employees and aid improvement in performance on an ongoing basis [13].

In order to re-configure health care systems to support people-centred care for chronic multimorbid conditions, there is a need to initially identify methods and practices that promote effective PM that can be harnessed to this end [14]. Methods refer to standard processes and procedures used by a PM system (this is usually prescribed by policy). Practices refer to the formal and informal application or execution of ideas, beliefs and methods. Such re-configured systems require a focus on training, motivation and readiness of health professionals who are at the forefront of facilitating changes in health care best practices, such as nursing staff who constitute the largest sector of health workers across the globe [15] (see Table 1 for definitions of PM methods and practices). As an important managerial tool, PM systems are a critical tool for facilitating health system reforms as they determine if health workers are working diligently, trained appropriately and adequately rewarded for providing quality health care interventions in line with the health systems reforms [16].

### Contribution to the field

#### PM methods and practices

PM systems are generally housed as part of role of human resource management (HRM), within the health care sector. The benefits of HRM practices to employee well-being and improved health outcomes have become a topical discussion among human resource practitioners and health care systems researchers around the world [17]. However, the impact of PM systems in health care settings has not received as much attention. While the nature of each health system and the use of HRM differ depending on national context, regardless of the context, it has become evident across national settings that HR is crucial in terms of its impact on patient outcomes and health care expenditures [18]. In order to determine how current health care delivery and reforms in health care systems may fully utilise HRM processes and systems such as a PM system to improve quality health care for people-centred care and promote better health outcomes [19], there is a need to initially examine evidence on PM methods and practices, as well as its consequences on the delivery of quality care among nurses in PHC settings.

#### PM opportunities and challenges

Some identified challenges include a world-wide shortage of nurses, health worker’s commitment and job satisfaction [8, 13–19]. These factors have an impact on patient care and the provision of quality service delivery. Generally, there is a limited understanding of how a PM system impacts on managing health workers, more specifically nurses and how it may be used to improve care delivery and ultimately patient outcomes. Researchers opine that the purpose of PM systems is to monitor employees’ performance, motivate staff through providing opportunities for skills development and improving morale through rewarding and incentivising good performance. Their argument is that a PM system is one of the most important components of HRM. It provides justifications for decisions regarding recruitment and selection, training and development needs of existing employees and how to optimise the quality of work and efficiency within individual health care centres as well as the health system in general [18]. Accordingly, a poorly implemented PM system can be detrimental to staff morale, overall job satisfaction and result in high staff turnover rates [20, 21]. The extent to which this has been investigated in health care settings is not clear. Some experts have varying opinions and approaches to PM systems that may add to HRM outcomes and quality of care [22]. Consequently, there is a need to review what is available on this topic for the purpose of creating a greater understanding of PM systems, as well as to identify knowledge gaps and providing recommendations on how future research may fill these gaps.

The aim of this scoping review was to systematically map the available evidence on the PM of nurses in PHC settings in order to enhance our understanding of the role of PM systems in improving ‘quality of care’, so as to understand how PM systems need to be strengthened for managing performance in PHC settings that encourages improving quality people-centred care and improve patients’ outcomes. This review offers a broad overview of managing the performance of nurses working at various PHC settings. In addition, it provides an analysis of international methods and practices used to manage nurses. From these methods, it is possible to identify best practices for suitable PM approaches.

The review of primary research has gained popularity, as evidence-based practices gain recognition as a benchmark for care and primary research sources continue to grow [23]. A scoping review is considered as a relatively new method for reviewing literature, with the first such framework published in 2005. This method of literature review is an advantage for synthesising research evidence and mapping existing literature in a given field in terms of its prevalence and key features. Hence, it is also referred to as a ‘mapping’ review [23, 24].

## Methodology

### Systematic scoping review

We will conduct a systematic scoping review of grey and peer-reviewed literature on PM and its influence on quality of care among nurses in PHC settings. The review will be guided by the Arksey and O’Malley’s scoping review framework [25], which outlines the following steps:

- Stage 1. Identifying the research question
- Stage 2. Identifying relevant studies
- Stage 3. Study selection
- Stage 4. Charting the data
- Stage 5. Collating, summarising and reporting the results

The recommendations of Levac et al. (2010) will be used to improve the transparency of each step pertaining to the conduct of the systematic scoping review [26].

#### Stage 1: Identifying the research question

The central research question of the study is as follows:

What is the existing evidence on the influence of PM methods and practices on quality of care among nurses in PHC?

The sub-research questions are as follows:

i. What are the common challenges and opportunities reported on various PM methods and practices?
ii. What are the key gaps in literature on the contribution of effective PM on quality of care among nurses in PHC settings?

The study will use the broad population, concept and context (PCC) framework recommended by the Joanna Briggs Institute for Scoping Reviews [27, 28]. The design of the search strategy will be underpinned by a key inclusion criteria (see Table 2).

**Table 2:** Pilot database search results

Date of search	Keyword search	No. of publications retrieved	Search engine utilised
30 April 2019	(nurses OR nurse practitioners OR registered nurses) AND (performance management OR performance appraisal OR performance reviews OR performance management and appraisal systems) AND (primary health care OR clinics)	696	PubMed

**Fig. 1:**
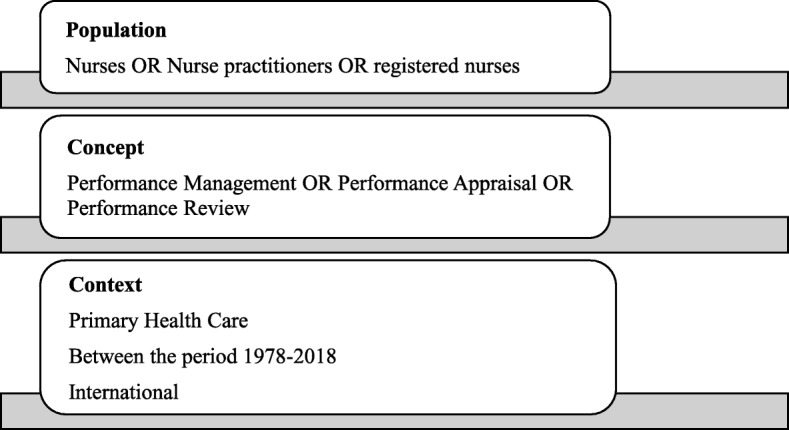
PCC Framework

The PCC framework to determine the research questions is illustrated in Fig. 1.

#### Stage 2: Identifying relevant studies

We will identify relevant studies by conducting a comprehensive search on the following electronic databases: *Academic Search Complete*, *PsycARTICLES*. *PsycINFO*, *Cumulative Index to Nursing and Applied Health Literature* (*CINAHL*), *Medline*, *Cochrane Library and PubMed*. Literature will include published peer-reviewed journal articles with primary studies which have a transparent empirical base utilising qualitative, quantitative and mixed method research design and grey literature addressing the research questions.

To achieve a comprehensive search, websites such as the *WHO* and *governmental websites* will be used to gather policies and guidelines on PM for the respective health care sectors. Databases such as Google *Scholar*, Union Catalogue of Theses and Dissertations (UCTD) via SABINET Online and WorldCat Dissertations and Theses via OCLO will also be used to find relevant literature.

A hand search through the main published texts used in PM systems and its outcomes will also be conducted. In addition, articles will be searched through the ‘cited by’ search as well as citations included in the reference lists of included articles. The search terms will include Nurse OR Nurse Practitioners OR Registered Nurse AND, Performance Management OR Performance Appraisal OR Performance Review OR Performance Management and Appraisal Systems AND Primary Health Care or Clinics. This search strategy was piloted to check the suitability of selected electronic databases and key words (see Table 2).

#### Stage 3: Study selection

Following the keyword search, relevant citations must be selected through title, abstract and full-text screening. The study selection process involves the elimination of studies that do not address the main research question. Developing an inclusion and exclusion criteria at the outset of the study ensures there are clear guidelines enforced, so each researcher is consistent in decision-making on the relevance for each citation [25]. An inclusion and exclusion criteria reduce the risk of bias in the review, thereby minimising the risk of error and promoting credibility of the findings.

In Table 3, information is provided about the inclusion and exclusion criteria that will be adhered to.

**Table 3:** Inclusion and exclusion criteria

Inclusion criteria	Exclusion criteria
• Be available in full text • Be in all languages • Studies that show evidence on performance management • Studies based on nurses or nurse practitioner or registered nurses • Must have been published between 1978 to date • Must be within the primary health care sector • All study design	• Studies with no evidence on performance management, appraisal or review • Studies published before 1978 • Studies not within the primary health care health sector • Studies not based on nurses or nurse practitioner or registered nurses

**Fig. 2:**
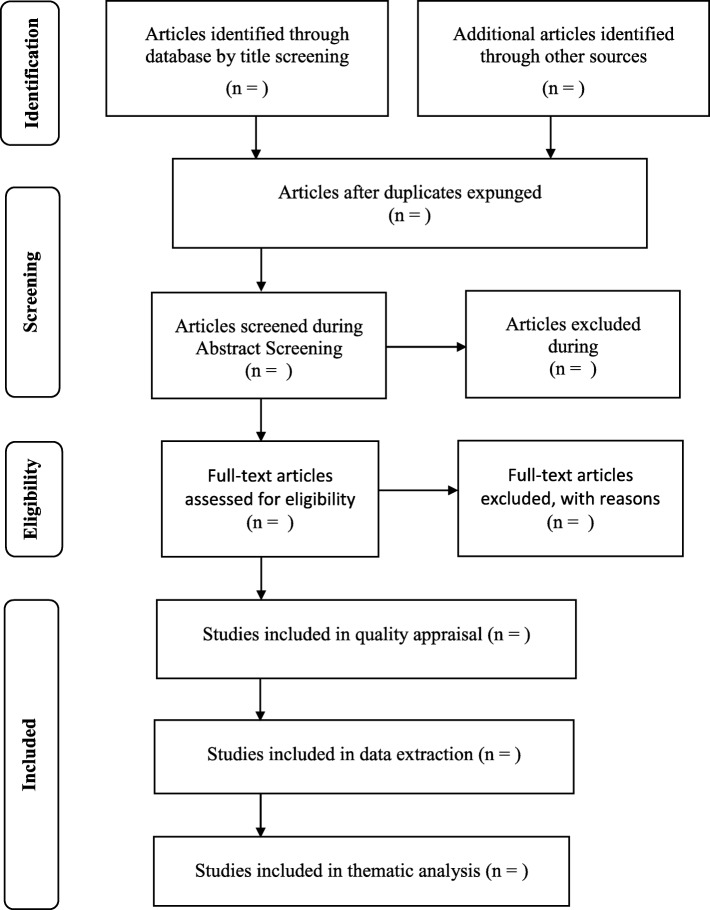
Example of PRISMA-ScR chart. Source: The PRISMA-ScR = preferred reporting item for systematic reviews and meta-analyses extension for scoping reviews [27, 29]

An Endnote™ library will be created for the aforementioned review. The primary investigator (CZM) will conduct a comprehensive database search and screen titles from the previously mentioned databases with the assistance from a senior librarian at the University of KwaZulu-Natal (UKZN) library services to assist with the search. All references screened will be exported to the Endnote library; title and abstract screening will be conducted. Once the initial screening is completed, eligible references are kept, and duplicates will be removed. The full text of eligible abstracts will be retrieved. To optimise the full article search procedure, the reviewers will further consult with the librarian to assist with locating and retrieving articles that will be included in the full article screening. In cases where the reviewers are unable to retrieve the articles from the databases, a request will be lodged with the relevant authors. Two reviewers (CZM and TS) will discuss eligible and ineligible studies to identify if there are any discrepancies [27]. Should the reviewers be unable to resolve disagreements through discussion, a third reviewer will be consulted (TPM-T). The screening results will be reported accordingly using the PRISMA chart as depicted in Fig. 2 [30, 31].

#### Stage 4: Charting the data

The process of extracting data aims to generate a descriptive summary of the results that corresponds to the aim and research question of the scoping review at hand. A draft data charting table (see Table 4) has been developed to facilitate the collection and sorting of key pieces of information from articles that have made the selection [32]. A data charting form, highlighting the important aspects for the study will be developed and piloted. The variables and themes included will answer each of the research questions. One reviewer will be involved in data extraction (CZM). Once completed, this process will be verified by the two other reviewers (TS and TPM-T). The data charting form will be updated as and when required.

**Table 4:** Data extraction/data charting tool

Author and publication year	
Type of publication	
Aims or research questions	
Study design	
Country	
Health care setting	
Study population	
Category of nurse	
Intervention	
Key findings	
Most significant findings	
Conclusions	
Additional comments	

#### Stage 5: Collating, summarising and reporting the results

To provide a narrative account of the data extracted from the included studies, data will be analysed using content and thematic analysis. Content and thematic analysis is useful as it provides a descriptive presentation of data. Through the identification of common themes in the text, the researcher is able to analyse the data. The data will be extracted around the following themes: PM initiatives, managing performance of nurses in PHC settings and the use of PM to influence the improvement of the quality of health care.

### Quality appraisal

The Mixed Method Appraisal Tool (MMAT) will be used to assess the quality of the studies [32, 33]. Each section is divided by research design type. During the appraisal process, the following will be used:

- Section 1 of the MMAT will be used to review the quality of a qualitative study;
- Section 2 is for quantitative randomised controlled studies;
- Section 3 will be used for non-randomised studies;
- Section 4 is for descriptive studies;
- Section 5 is for mixed-method research methodology studies.

[Note: for a mixed methods study, we will use section 1 for appraising the qualitative component, the appropriate section for the quantitative component (2 or 3 or 4) and section 5 for the mixed methods component]. This tool is valuable in examining the suitability of an objective of a study, its methodology, the appropriateness of the study design, the data collection, the study selection, the data analysis, the findings presentation as well as the discussion and conclusion. The results from the scrutiny of the above-mentioned aspects will determine the quality of the articles and if the studies will be included after the extraction of the data [33]. The quality of the articles will be graded per domain on a percentage basis. For qualitative (QUAL) and quantitative (QUAN) studies, the grading of each study will be based on the number of criteria met divided by 4, the score ranging from 25 (*only one criterion was met) to 100% (****all criteria were met). For mixed methods (MM) studies, the quality of the combination cannot exceed the quality of the weakest component. Therefore, the overall quality score is the lowest score of the study components. Thus, the score of 25% (*) is gained when QUAL = 1 or QUAN = 1 or MM = 0, 50% (**) when QUAL = 2 or QUAN = 2 or MM = 1, it is 75% when QUAL = 3 or QUAN = 3 or MM = 2 and it is 100% when QUAL = 4 or QUAN = 4 and MM = 3. For the purpose of this study, 25% is considered low quality, and above 80% is considered high [31, 34]. Grey literature will be assessed using the Joanna Briggs Institution (JBI) Narrative, Opinion, Text Assessment and Review Instrument (NOTARI) systematic reviews. Using the JBI Reviewer’s Manual 2014, any issues relating to the including suitability of topic selection, critical appraisal, data extraction and synthesis will be addressed. Textual evidence requires three levels of credibility. Therefore, the reviewers are required to determine if, when comparing the conclusion with the argument, the conclusion represents evidence that is Unequivocal (U) (relates to evidence beyond reasonable doubt), Credible (C) and Unsupported (findings that are not supported by the data) [35].

## Discussion

PM systems are a significant element of HRM. The growing need for improved clinical outcomes and quality of care has highlighted the importance of standards of care and managing the performance of health workers. However, poor practices in the implementation of PM systems within the health sector have been shown to have a negative impact on employees’ perceptions of fairness and accountability, which in turn leads to high staff turnover and poor clinical outcomes. Literature on the PM of nurses in health care is abundant. With the shift towards PHC and its well-documented benefits, the reviewers will aim to map literature around the evidence, preferences and practices of the PM of nurses, in light of the need to ensure health workers are adequately trained and rewarded for meeting the needs of existing health care systems. Enhancing methods and practices of PM will help inform decisions on how the practice of people-centred care may be improved, by ensuring good performance is rewarded and health workers are equipped with tools that assist and facilitate effective chronic care practices in PHC settings.

The reviewers anticipate this scoping review finding will assist in mapping evidence of best practices and preferences on PM methods and practices. Likewise, the reviewers hope to expose knowledge gaps and limitations, as well as inform future research. Findings will be disseminated electronically, in print, through peer presentations and conferences on strengthening health systems, HRH or conference proceedings, symposia and other research contributions that examine investing in health care human capital.

## Data Availability

All data generated or analysed during this study will be included in the published systematic scoping review article and will also be made available upon request.

## Abbreviations

HCW: Health care workers
HR: Human resource
HRH: Human resources for health
HRM: Human resource management
HRP: Human resource practitioner
MMAT: Mixed Method Appraisal Tool
NCD: Non-communicable disease
PA: Performance appraisals
PCC: Population concept context
PHC: Primary health care
PM: Performance management
PRISMA-ScR: Preferred Reporting Items for Systematic Reviews and Meta-Analyses extension for scoping reviews
QI: Quality improvement
QOC: Quality of car
TA: Thematic analysis
UKZN: University of Kwa-Zulu Natal
WHO: World Health Organization
